# Scene Uyghur Text Detection Based on Fine-Grained Feature Representation

**DOI:** 10.3390/s22124372

**Published:** 2022-06-09

**Authors:** Yiwen Wang, Hornisa Mamat, Xuebin Xu, Alimjan Aysa, Kurban Ubul

**Affiliations:** 1 School of Information Science and Engineering, Xinjiang University, Urumqi 830046, China; wywpure@stu.xju.edu.cn (Y.W.); hornisamamat@xju.edu.cn (H.M.); xuxuebin@xju.edu.cn (X.X.); 2 Xinjiang Key Laboratory of Multilingual Information Technology, Xinjiang University, Urumqi 830046, China

**Keywords:** natural scene image, Uyghur text detection, multi-oriented text, fine-grained feature representation, adaptive spatial feature fusion

## Abstract

Scene text detection task aims to precisely localize text in natural environments. At present, the application scenarios of text detection topics have gradually shifted from plain document text to more complex natural scenarios. Objects with similar texture and text morphology in the complex background noise of natural scene images are prone to false recall and difficult to detect multi-scale texts, a multi-directional scene Uyghur text detection model based on fine-grained feature representation and spatial feature fusion is proposed, and feature extraction and feature fusion are improved to enhance the network’s ability to represent multi-scale features. In this method, the multiple groups of 3 × 3 convolutional feature groups that are connected like the hierarchical residual to build a residual network for feature extraction, which captures the feature details and increases the receptive field of the network to adapt to multi-scale text and long glued dimensional font detection and suppress false positives of text-like objects. Secondly, an adaptive multi-level feature map fusion strategy is adopted to overcome the inconsistency of information in multi-scale feature map fusion. The proposed model achieves 93.94% and 84.92% F-measure on the self-built Uyghur dataset and the ICDAR2015 dataset, respectively, which improves the accuracy of Uyghur text detection and suppresses false positives.

## 1. Introduction

**Figure 1:**
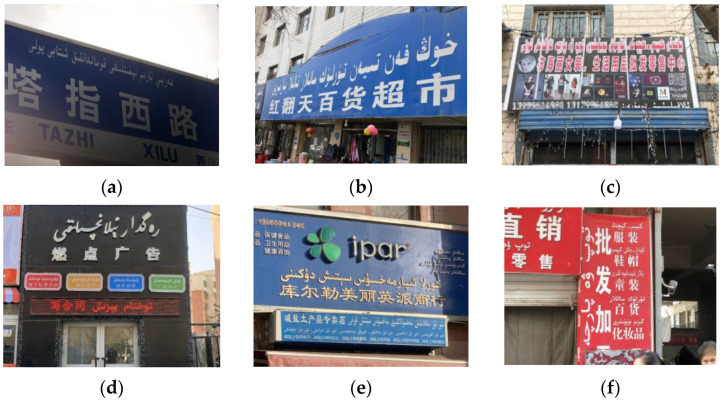
Difficult detection text sample. (**a**) Scene image with uneven illumination; (**b**) perspective text in scene image; (**c**) scene image with partial text obscured; (**d**) artistic text in scene image; (**e**) multiscale text in scene images; and (**f**) multidirectional text in scene images. (Text description in the figure: (**a**) Tazhi West Road; (**b**) Hongfantian department store; (**c**) Buhaili women’s clothing, daily necessities wholesale and retail center; (**d**) Burning advertisement; (**e**) korla ipar store, which sells alkaline salt products; (**f**) This is a store that wholesales or retails clothing, cosmetics, etc.)

In Xinjiang Uyghur Autonomous Region of China, Uyghur, a treasure of ethnic minorities, is still the common language of most Uyghur compatriots. It records the time-honored traditional customs and precious historical culture of the Uyghur. In natural scenes, texts are more general and logical, which can intuitively convey high-level semantic information and help humans analyze the content of the scene [1]. At present, the academic research of Uyghur text detection algorithms is still in its infancy. As a front-end task of character recognition, scene text detection is of great significance in the fields of electronic ancient book protection [2,3], intelligent transportation [4,5], etc. Modern Uyghur adopts an improved Arabic alphabet, which contains 32 basic letters and is written from right to left. Due to the difference in the position of each basic letter before and after the composition of words, the same letter will also have multiple variants such as single, front, double, and back. Compared with common language texts, Uyghur words have a larger scale change. In addition, the main structure of some letters is the same, but different letters are distinguished by the number of upper and lower points. These upper and lower points are easily recognized as background noise during detection, resulting in missed detection. Furthermore, extreme-scale text often exists in the same scene image, and it is often difficult to detect the scene text image due to the complex and changeable natural environment conditions and shooting angle and distance. In Figure 1, we show six examples of text images with different scene conditions. As follows, Figure 1a–f, respectively, show text examples in complex scenes with uneven illumination, perspective text, partial occlusion, artistic font, multi-scale text and multi-angle tilt. The displayed images are from the scene Uyghur text image dataset built in this paper.

First of all, a major difficulty in the research of scene Uyghur text detection is that considerable results have been achieved in the research of scene text algorithms in major languages such as English and Chinese. However, there are few studies on Uyghur text detection, and there is a lack of public scene Uyghur image datasets in academia. Some researchers have tried to use synthesis methods to embed Uyghur characters in web images, but in some synthetic images, Uyghur characters exist as a single character, which loses the stickiness of Uyghur characters, and most synthetic image characters are of the same size, without scale randomness, which is quite different from the real scene.

In addition, another difficulty in scene Uyghur text detection is that the scene image text has a changeable shape and complex backgrounds, and often contains objects and trademarks similar to the fonts, which is easy to cause false detection, and the Uyghur words are long, and the characters also contain small upper and lower points, which means it is easy to cause missed detection. At the same time, Uyghur words in the same street view image will show strong scale changes and text angle inclinations. Therefore, there is a high requirement for the expressive ability of the network with multi-scale features to correctly distinguish text targets from complex background noise. At present, most researchers use smaller convolution kernel [6] and residual structure [7] to stack more convolution layers to effectively enlarge the receptive field and improve performance, but lose more of the original image details, which has a great impact on the detection effect of small-scale text. The main contributions of this paper are summarized as follows:

(1) First of all, aiming at the lack of public Uyghur text image datasets in academia, this paper constructs a Uyghur text image dataset in natural scenes, and annotates it at the word level. The dataset contains 4000 images including store signs, road signs and other street scenes.
(2) Since there are texts of extreme sizes in the same scene image, and the text occupies a small proportion of the area in the image, the feature information of multi-scale objects in the perception context is of great help for understanding local text objects. It is beneficial to identify false detections of objects similar to text texture features. In this paper, a text feature extraction network based on fine-grained feature representation is proposed for the multi-scale text and the false positives of text-like objects. The network gradually increases the receptive field by embedding multi-level convolutions in the residual structure, which can increase the number of scales represented by the output feature in a more fine-grained form, acquire the contextual information of the text instance while capturing the features, and thus improve the efficiency of handling Uyghur text instances with extreme scale distribution and effectively suppress the false positives.
(3) Considering that the scales of text objects mapped by different levels of feature maps in the Feature Pyramid Network (FPN) are different, and this inconsistency will lead to the conflict of information between different feature scales when the features of each level are fused. In order to reduce the interference of inconsistency on feature learning, this paper proposes a fusion strategy based on multi-level feature weighting, which eliminates the information conflict between positive and negative samples caused by fusion by calculating the spatial weight of multi-level feature maps.

This paper is organized as follows:

Section 2 discusses the current research status of classical scene text detection algorithms for common languages and Uyghur, respectively. Section 3 focuses on the general framework of the method proposed in this paper, and introduces the principle of Res2Net module and AMF strategy in detail. Section 4 shows the text detection results, and the experimental data are evaluated and discussed. The advantages and disadvantages of the experimental results are discussed in Section 5. Section 6 concludes and prospects the work of this paper.

## 2. Related Works

With the development of the scene text detection algorithm, the method based on deep learning breaks the limitations of traditional feature design and obtains higher detection accuracy [8]. According to the difference of the text bounding box inference process, the existing scene text detection models are roughly divided into two methods: segmentation-based and regression-based. The regression-based scene text detection algorithms [9,10] are usually similar to the common object detection algorithm. Firstly, the convolutional networks are used to extract image features. Then, multiple anchor boxes with different length and width scales are preset for each pixel on the convolutional layer using methods such as the Region Proposal Network [11], to determine whether the anchor box contains a text area, and then step by step through iterative learning. The candidate text boxes are subjected to regression correction, and the non-maximum suppression algorithm discards and retains redundant text prediction boxes according to the threshold to obtain the final text box results. Ma et al. [12] proposed an RPN structure with rotation angle information to better adapt to the detection of inclined text. Tang et al. [13] proposed the bottom-up detection method Seglink++, which can simultaneously predict the text box components and the attractive and repulsive connection relationship between the components, which solves the detection problem of text boxes with extreme aspect ratios. Long et al. [14] proposed to describe irregular text by a series of ordered disks that overlap each other on the text centerline. The method predicts the score map of text centerline, text area, and geometric attributes such as disk radius and angle based on the FCN network, which further solves the problem of wrapping tightness of curved text boxes. The single-stage detection network EAST proposed by Zhou et al. [15] directly predicts the score map and the pixel-to-boundary distance and angle, which reduces the redundant process of the multi-stage algorithm and greatly improves the detection speed, but the algorithm has poor detection effect for slender text. Further, [16] proposed MOST, which adopts the changeable convolution to dynamically adjust the positioning prediction according to the shortcomings of the EAST network, uses the position-sensitive map up, down, left, and right, and adopts the non-maximum suppression algorithm to solve the problem of extreme aspect ratio text detection. In [17], the improved YOLOv3 algorithm is proposed to use the sliding vertex text box and MD-loss to improve the accuracy of multi-directional text detection. In paper [18], FCENet is proposed to fit the contours of arbitrary-shaped text boxes in the Fourier transform domain, making the inference process of text boxes simpler. Zhao et al. [19] proposed a particle swarm optimization algorithm to simulate text shape, and designed an instance-aware network to combine text components, which adapt to the detection of irregular text instances.

As the regression-based detection method needs to preset a variety of anchor boxes in advance, it has certain limitations for the detection of extreme scales and irregular text areas. The segmentation-based text region location algorithm uses the idea of semantic segmentation to classify the image at the pixel level. Firstly, this kind of method uses a convolutional neural network to extract rich semantic information from scene text image, then classifies text and background pixels at the pixel level to obtain the text region segmentation maps, and finally aggregates pixel inferences belonging to the text regions through post-processing algorithms out the prediction text box [20,21]. As the segmentation detection algorithm has no limitation of a preset anchor box, it is good for detecting long text and irregular shape text. Deng et al. [22] proposed the text instance segmentation network PixelLink, which predicts the text region by pixel-by-pixel prediction of a text score map and its probabilistic connections with surrounding pixels in eight directions. In paper [23], SEMPANet is based on the PANet algorithm, introducing the SE module to select beneficial feature channels, and at the same time introducing the MPA module, which makes the bottom-up information easier to propagate to the upper layers and improves the network performance. In [24], the TextField method proposes a concept of a direction field represented by a two-dimensional vector image, which uses a direction field to encode binary text mask and direction information that can be used to separate adjacent text instances, which is conducive to the detection of irregular text. In paper [25], Qin et al. Improved Mask R-CNN and proposed a full connection structure with non-shared weights, which uses global information to distinguish text regions. In [26], the TextBPN method uses the encoder composed of GCN and RNN to learn the topology of the text area, and iterative refinement solves the problem of noise in the outline of the text box. Liao et al. proposed the segmentation-based algorithm DBNet [27], which simplifies the post-processing algorithm by using the differentiable binarization of pixels and enhances the robustness of binary graphs, but it lacks the use of multi-scale feature representation and spatial information, which limits its text region positioning ability.

At present, the text detection methods in common languages have achieved ideal results, while the research on Uyghur text detection is still in the development stage. Yan et al. [28] proposed FASTroke algorithm for stroke extraction according to the difference between the color, texture and background of Uyghur characters, and introduced a component-level classifier to reduce the false detection of text instances. Its comprehensive evaluation index F-measure for Uyghur text line detection can reach 83%. Based on a fully convolutional network, Fang et al. [29] used three RPNs to directly generate text-line-level candidate boxes so that the network could learn the baseline features of Uyghur texts, and at the same time, it could reduce the errors caused when words were merged into sentences, and improve the detection accuracy. Its comprehensive index can reach 85.5%.

## 3. Methods

**Figure 2:**
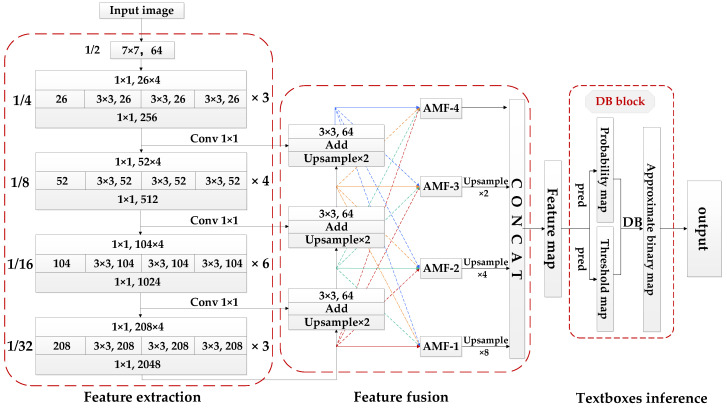
The framework of the proposed text detection model. It includes a Res2Net backbone network for feature extraction; an AMF feature fusion module and a text box reasoning module based on DB method.

Based on DBNET, this paper segmented the text instance at the pixel level and converted the predicted probability graph to the binary graph by differentiable binarization threshold to obtain the final text box. The detection process was divided into three steps: feature extraction, feature fusion and text box reasoning. The proposed text detection method focuses on feature extraction and feature fusion. Firstly, based on the Res2Net [30] structure, this paper constructs multi-level residual class connections in a separate bottleneck structure, and convolutes with detailed division to expand the receptive field range of each submodule and capture the multi-scale features of the image. Secondly, the spatial feature fusion algorithm [31] based on a feature pyramid network [32] is used to eliminate the conflict of positive and negative sample information between multi-scale feature maps by calculating the weight of spatial features, so that the feature maps to be detected have more detailed positioning information and semantic information. The structure of the multi-directional scene Uyghur text detection model proposed in this paper is shown in Figure 2.

### 3.1. Fine-Grained Feature Extraction Module

Generally, the text area occupies a small proportion of the natural scene images, mostly narrow quadrilaterals, and the Uyghur text symbols have strong scale changes. Therefore, the designed feature extraction network can describe the multi-scale representation of text using a wide receptive field, which is very important to distinguish text objects from complex background noise.

**Figure 3:**
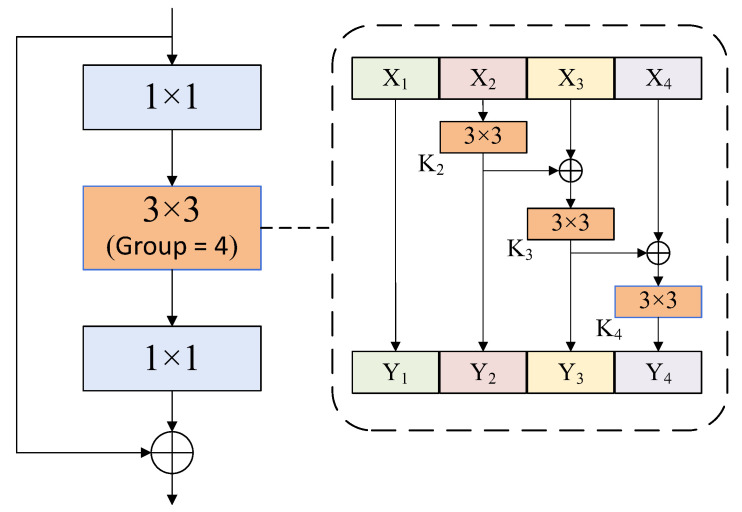
Hierarchical architecture of bottleneck blocks. On the left is the bottleneck block structure in the original residual network. In this paper, the intermediate convolution of the standard bottleneck structure is replaced by the multi-level convolution group on the right.

Different from the defects of residual network and other networks that use a large receptive field, which makes the details of low-resolution feature maps relatively rough, and thus has poor detection effect on small text objects. In this paper, the Res2Net network is introduced to enhance the receptive field. The algorithm redesigns the bottleneck structure based on the overall framework of ResNet50, which replaces the 3 × 3 convolution in the standard bottleneck structure with the 3 × 3 convolution group based on multi-level residual architecture to increase the receptive field inside the residual block, to obtain finer-grained scale features of different levels in the image. Its network structure is shown in Figure 3.

Firstly, the output feature map after 1 × 1 convolution is evenly divided into *s* feature map subsets in the channel dimension. The larger the scale dimension is, the richer its receptive field, the more features it learns, and the stronger its representation ability for multi-scale text areas. In this experiment, *s* is set as 4, 6, and 8, respectively, for comparison. The experimental results are shown in Section 4. We denote the feature subsets as *X_i_*, *i*∈{1, 2,…, *s*}, which has the same size, but the channel number is 1/*s* of the input feature map. In the experiment, the channel width of the feature subset is set to 26. In order to reuse part of the original features and reduce the parameter calculation, *X*_1_ is not convoluted, and each remaining *X_i_* corresponds to a convolution of 3 × 3, defined as *K_i_*(), whose output is recorded as *Y_i_*. The feature subset *X_i_* is added with the output of *Y_i_*_−1_() and then fed into *K_i_*(). *Y_i_* can be described as follows:

(1) $$Y_{i}=\left\{ \begin{array}{ll} X_{i} & i=1\\ K_{i}\left(X_{i}\right) & i=2\\ K_{i}\left(X_{i}+Y_{i-1}\right) & 2<i\leq s \end{array}\right.$$

Then, connect the output *Y_i_* of each stage using concat function and finally send it to 1 × 1 convolution. Each level of the 3 × 3 convolution operation can fuse the feature information of all the previous channels so that each Res2Net can obtain a different number of feature combinations and different receptive field sizes, which can improve the ability of the whole model detection and segmentation of objects in the image with a more fine-grained feature description.

### 3.2. Adaptive Multi-Level Feature Fusion Module

Since the feature fusion network FPN is used to detect text, large text instances are usually mapped in the low-resolution feature map and small text instances are mapped in the high-resolution feature maps. When a positive sample is detected in the feature map of one level, the corresponding area of other levels may just be the background area, which will lead to the influence of both positive and negative samples in the gradient when the FPN algorithm performs backpropagation. Therefore, when the image includes large and small objects at the same time in the training process, the information in the feature map of each layer of the pyramid structure is not synchronized with each other, which will adversely interfere with the gradient transmission and affect the detection effect.

**Figure 4:**
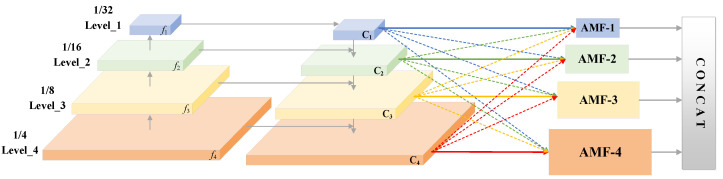
Adaptive multiscale feature fusion framework.

As shown in Figure 4, in the process of feature fusion, the features of different levels are first input into the pyramid structure, and the feature maps are up-sampled from the feature map C_1_ with the lowest resolution in turn, and the horizontal 1 × 1 convolution is performed. Add the same size *f_i_* to obtain four levels of fusion features C_1_, C_2_, C_3_ and C_4_, with sizes of 1/4, 1/8, 1/16 and 1/32 of the original image, respectively. Then, according to the adaptive multi-level feature fusion method, the size adjustment and adaptive fusion of each feature map are carried out, respectively. In the first step, feature map size adjustment refers to adjusting the resolution of the feature map on other different levels *n* (*n* ≠ *l*) by using down-sampling based on pooling and convolution and up-sampling based on interpolation for the specified *l*-layer, so that it has the same scale size as the feature map *X_l_* of the specified *l*-layer. The up-sampling strategy uses a 1 × 1 convolution to adjust the channel dimension of the feature map to make it consistent with level *l,* and then obtains the feature maps with different resolution multiples by adjusting the spatial size scaling factor in the nearest neighbor interpolation method. For down-sampling, a 3 × 3 convolution with stride of 2 is used to compress the feature map size to 1/2 of the original feature map, and the channel dimension is adjusted at the same time. For the down-sampling of 1/4 ratio, a maximum pooling is performed first, and then a 3 × 3 convolutional layer with a stride of 2 is used to further reduce the image resolution. For the 1/8 scale, it is obtained by performing two convolutions after the pooling layer. In the second step, we will adaptively fuse the feature maps of each level adjusted according to the target size. The fusion calculation formula is as follows:

(2) $$y_{ij}^{l}=\alpha_{ij}^{l}x_{ij}^{1\to l}+\beta_{ij}^{l}x_{ij}^{2\to l}+\gamma_{ij}^{l}x_{ij}^{3\to l}+\mu_{ij}^{l}x_{ij}^{4\to l}$$

where $x_{ij}^{n\to l}$ represents the feature vector at (*i*, *j*) of the feature map adjusted from layer *n* to layer *l*, and $x_{ij}^{1\to l}$, $x_{ij}^{2\to l}$, $x_{ij}^{3\to l}$, $x_{ij}^{4\to l}$ represent the feature map after scaling from levels 1, 2, 3 and 4, respectively. *α*, *β*, *γ*, *μ* are the weight parameters of four levels, which are obtained by convolution calculation of the feature map after adjusting the size, and after the four parameters are connected by concat function, softmax calculation is carried out to normalize the weight value to the range of [0, 1], and the sum of the four weights is 1. The normalization calculation is shown in Equation (3):

(3) $$\alpha_{ij}^{l}=\frac{e^{\lambda_{\alpha_{ij}}^{l}}}{e^{\lambda_{\alpha_{ij}}^{l}}+e^{\lambda_{\beta_{ij}}^{l}}+e^{\lambda_{\gamma_{ij}}^{l}}+e^{\lambda_{\mu_{ij}}^{l}}}$$

where $\lambda_{\alpha_{ij}}^{l}$, $\lambda_{\beta_{ij}}^{l}$, $\lambda_{\gamma_{ij}}^{l}$, $\lambda_{\mu_{ij}}^{l}$ are the control parameters. The feature fusion strategy based on adaptive multi-level weighting can adaptively combine all levels of features according to the weight of each layer on each scale, so that the network can directly learn the method of filtering positive and negative sample conflicts in the spatial dimension, set the weight of the corresponding background level to 0, and only retain the effective information for aggregation to improve the scale invariance of features. Finally, concatenate the fused feature maps {*Y*_1_, *Y*_2_, *Y*_3_, *Y*_4_} of the four levels to obtain the feature map for predicting the text border, which is 1/4 of the size of the original map.

### 3.3. Text Bounding Boxes Inferencing

In this paper, the improved segmentation-based method DBNet is used to infer the text position in the complex scene images, and the feature map is predicted through training and learning to obtain a threshold map and a probability score map, then an approximate binary map is calculated according to the score map and threshold map, and finally we obtain the text bounding box.

**Figure 5:**
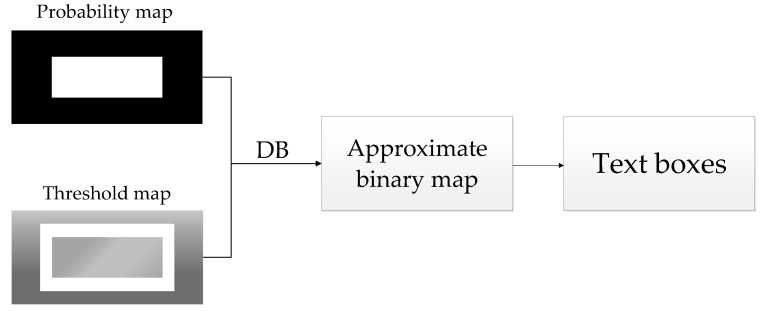
The flow chart of textbox inference.

As the binary image obtained by the traditional preset fixed threshold has the disadvantage of non-differentiability, the DB algorithm uses the calculation method of an approximate step function to adaptively binarize each pixel in the image. As can be seen from Figure 5, through threshold learning and differentiable operation, the threshold transformation is put into the network for training to obtain a more robust binary image, which can better distinguish the text area and background while simplifying the post-processing. The approximate binary mapping formula of the (*i*, *j*) position is as follows:

(4) $${\hat{B}}_{i,j}=\frac{1}{1+e^{-k(P_{i,j}-T_{i,j})}}$$

where *P_i,j_* and *T_i,j_* represent the pixel values at the positions of probability map and threshold map (*i*, *j*), respectively, and k represents the magnification factor, which is set to 50.

The segmentation of text area and background noise is essentially a binary classification task. When binary cross-entropy is used as its classification loss, the DB function is shown in Equation (5):

(5) $$f(x)=\frac{1}{1+e^{-kx}}$$

where *x* = *P_i,j_* − *T_i,j_*, (if *x* > 0, it is a positive sample, otherwise it is a negative sample), the loss functions *l*_+_ and *l*_−_ of positive and negative samples and their partial derivatives to input x are shown in Equations (6) and (7), respectively.

(6) $$\begin{array}{l} l_{+}=-\log\frac{1}{1+e^{-kx}}\\ l_{-}=-\log(1-\frac{1}{1+e^{-kx}}) \end{array}$$

(7) $$\begin{array}{l} \frac{\partial l_{+}}{\partial x}=-kf(x)e^{-kx}\\ \frac{\partial l_{-}}{\partial x}=kf(x) \end{array}$$

In the training process, the prediction of probability map and the generation of binary map are supervised by the same label. The generation method is as follows: shrink the label G of the original box inward by *D* offset distance to obtain the shrinkage box G_s_. We label the pixels in the G_s_ box as 1 and the pixels outside the box as 0. The offset is calculated as follows:

(8) $$D=\frac{A(1-r^{2})}{L}$$

Firstly, expand the *D* distance of the original label g outward to obtain the expansion frame G_d_, calculate the minimum value of the distance from the pixel to each edge in the area from G_s_ to G_d_, divide it by the offset *D*, normalize it, and then subtract the value with 1 to obtain a value in the interval of [0, 1], that is, the pixel label. Since the label of threshold graph t cannot be 1 or 0, scale the value from 1 to 0.7 and expand from 0 to 0.3.

Finally, a threshold of 0.2 can be used for the approximate binary graph or probability graph to obtain the text-connected domain, and then the connected domain can be enlarged according to Equation (9) to obtain the final predicted text bounding box.

(9) $$D^{\prime }=\frac{A^{\prime }\times r^{\prime }}{L^{\prime }}$$

where, *r*′ is a super parameter, set to 1.5, *L*′ represents the perimeter of the connected domain, and *A*′ represents the area of the connected domain.

In this paper, binary cross-entropy loss is used as probability map loss *L_s_* and binary map loss *L_b_*, and the difficult case mining training strategy with a positive and negative sample ratio of 1:3 is adopted. The calculation formula is as follows:

(10) $$L_{s}=L_{b}=\sum_{i\in S_{l}}y_{i}\log x_{i}+(1-y_{i})\log(1-x_{i})$$

*L*_1_ distance loss is used as the loss function *L_t_* of the threshold diagram, and the calculation process is shown in Equation (11):

(11) $$L_{t}=\sum_{i\in R_{d}}|y_{i}^{*}-x_{i}^{*}|$$

The total training loss *L* is shown in Equation (12):

(12) $$L=L_{s}+\alpha L_{b}+\beta L_{t}$$

where parameter *α* set to 1, *β* set to 10.

## 4. Results

### 4.1. Datasets

**Figure 6:**
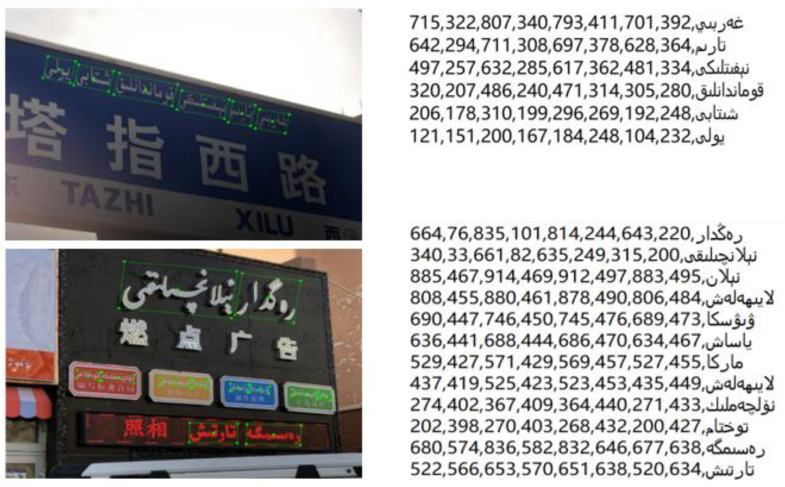
Annotation example of Uyghur dataset. The image on the left is the annotation box of visual Uyghur words, and the image on the right is the coordinates of the four vertices of the annotation text box and the corresponding text content. (Text description in the figure: Tazhi West Road and Burning advertisement.)

In this paper, the database adopts the open English data set ICDAR2015 [33] of text detection and recognition competition and the Uygur text detection data set of complex scenes collected locally. ICDAR2015 dataset contains 1500 scene images, including 1000 for training and 500 for testing. These images are captured by Google glasses at a relatively low resolution. The dataset contains a large number of small and fuzzy text instances, which are tilted at various angles, and provide annotations in the form of words. The self-built Uyghur scene image dataset contains 4000 multi-angle images. According to the ratio of 6:2:2, it is divided into 2400 image training sets, 800 image verification sets and test sets. These images are collected in the form of shooting, including advertising banners, road signs, store signs and other street scenes. Due to different shooting angles, distances and other environments, the text in the image has the characteristics of multi-scale and multi-directional tilt. In addition, these texts are marked at the Uyghur word level, and the labels are the horizontal and vertical coordinates of the four clockwise vertices of the text box and the text content saved in txt format. The annotation of the Uyghur data set is shown in Figure 6.

**Figure 7:**
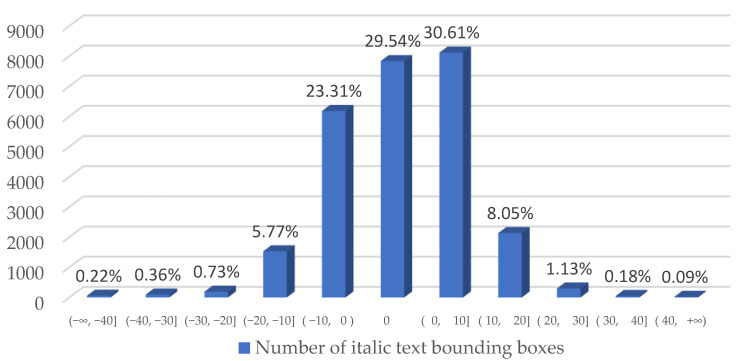
Proportion of text bounding boxes with different tilt angles. In this paper, the tilt angle of the text box is divided into 11 intervals with an interval of 10 degrees, ‘+’ represents the tilt of the text box to the left and ‘−’ represents the tilt to the right.

**Figure 8:**
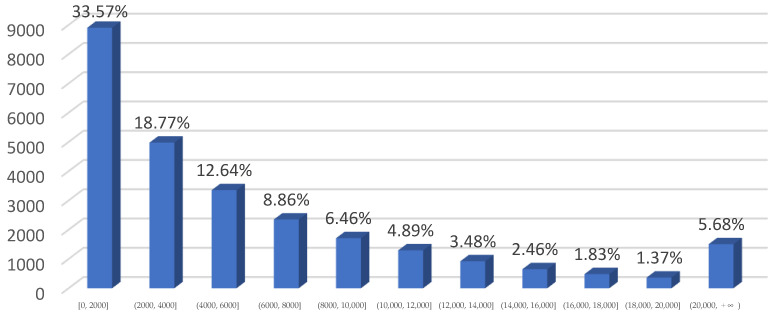
Proportion of text bounding boxes with different text area. In this paper, the area value of pixel level in label text box is used to reflect the size of text scale. The pixel area of text box is divided into 11 intervals with an interval of 2000 square pixels for quantitative statistics. The area unit is pixel^2^.

According to the text area characteristics of the self-built scene Uyghur dataset, this paper makes quantitative statistics on the tilt angle of the multi-directional text box and the area of multi-scale text box, respectively. The statistical results are shown in Figure 7 and Figure 8, respectively.

As can be seen from the above statistical Figure 7, in the self-built scene Uyghur dataset, 83% of the text boxes are in the range of 10° left and right tilt, 13.82% of the multi-directional text boxes are in the range of 10° to 20°, and nearly 4% of the text boxes are in the range of large tilt, and the tilt angle exceeds 20°. Generally speaking, the tilt angle of the text boxes in the self-built scene Uyghur dataset is basically in the range of 40° left and right tilt. A few text instances have a tilt angle greater than 40 degrees.

In Figure 8, the text box in the self-built Uyghur text dataset has a largescale span, a very small area and a high degree of two-level differentiation. The text box with an area of less than 2000 square pixels accounts for 33%, the text box with an area of more than 14,000 square pixels accounts for 11.34%, and 5.68% of the text box has a scale of more than 20,000 square pixels. It can be seen that the self-built Uyghur text data set has a high challenge for the detection of extreme size text.

### 4.2. Evaluation Criteria and Implementation Details

In the experiment, precision, recall, F-measure and Frames Per Second (FPS) are used to evaluate the text detection results. To determine whether the prediction is correct by comparing the Intersection Over Union (*IOU*) value of the predicted text box with the corresponding label box is greater than the set threshold value, which is set to 0.5. The calculation formula is as follows:

(13) $$IOU=\frac{pred\cap gt}{pred\cup gt}$$

where *pred* represents the area of the predicted text box and *gt* represents the area of the label text box. The number of real text boxes predicted as text boxes are recorded as *TP*, the number of real text boxes predicted as background areas are recorded as *FP*, the number of background areas predicted as text boxes are recorded as *FN*, and the number of background areas predicted as the background are recorded as *TN*, then the calculation formula of comprehensive values of precision, recall and F-measure are as follows:

(14) $$P=\frac{TP}{TP+FP}$$

(15) $$R=\frac{TP}{TP+FN}$$

(16) $$F=\frac{2\times P\times R}{P+R}$$

In addition to evaluating the precision of text detection, having a fast detection speed is also very important for real-time text detection. In this paper, the FPS index is used to evaluate the detection speed of the algorithm, that is, the number of pictures that can be processed per second.

The video card model of the experimental computer in this paper is a single NVIDIA GeForce RTX 2080ti graphics card with 11 G video memory; Intel(R)Core(TM)i9-7980XE CPU, 2.6 GHz and 36 GB of memory; the system is Ubuntu 18.04; we use the Pytorch framework to compile the code with Python 3.7; and the compiler is Pycharm of community version 2021.1. In the process of the experiment, firstly, the pretraining model of the Res2Net network on the ImageNet dataset is used as the initialization parameter of the feature extraction network. Before training, the image is preprocessed, and its size is adjusted to 640 × 640. The training batch size is set to 4. During the training, we use the WarmupPolyLR strategy to reduce the learning rate. The warmup epoch is set to 3; the initial learning rate is set to 0.001, and AdamW optimizer is used.

### 4.3. Selection Strategy of Hierarchical Scale “s” of Residual Block and Ablation Study

Based on the DBNet text detection algorithm, this paper improves the feature extraction and feature fusion module. To evaluate the comprehensive performance of the model, according to the proposed Res2-AMF text detection model, aiming at the hierarchical strategy of bottleneck blocks and improved modules in a residual network, experiments are carried out on public data sets and self-built Uyghur datasets, and the experiments all use the same evaluation index.

#### 4.3.1. Selection Strategy of Hierarchical Scale “s” of Residual Block

In this paper, the selection strategy of the scale s of the convolution integration layer in the bottleneck structure in the feature extraction network is tested. In the experiment, s is set to 4, 6, and 8, respectively. The results are shown in Table 1.

It can be seen from Table 1, on the two datasets, the precision, recall and F-measure indexes of the model are improved with the increase of the hierarchical scale s. Compared with s = 4, when s is set to 6, the F-measure of model is improved slightly, but on the public English dataset ICDAR2015 and the self-built Uyghur dataset, the detection speed FPS is reduced by 2.88 frames per second and 4.58 frames per second, respectively. When s is set to 8, the indexes of P, R and F are improved by 1.87%, 0.34%, and 1.05%, respectively, on the ICDAR2015 dataset, but the detection speed decreases by 6.16 frames per second. The combined metrics improve by 0.65% on the Uyghur dataset, but there is a significant decrease in speed of 10.1 frames per second. It can be seen that the larger the control parameter s is, the number of channels divided increases, so that each level of feature map has richer receptive fields, and its multi-scale representation ability is stronger, but with the increase of the middle 3 × 3 convolution number of residual blocks, the detection rate also decreases significantly. Therefore, the hierarchical scale of the residual block is set to 4 in the experiment.

#### 4.3.2. Ablation Study

Aiming at the two modules of fine-grained feature extraction network and adaptive multi-level feature map fusion network, ablation experiments are carried out on public data sets and self-built Uygur data sets. To verify the effectiveness of the module proposed in this paper, a network model using the Res2Net module, AMF module and two modules at the same time is designed, which is compared with the baseline method DBNet based on the segmentation method. The experimental results are shown in Table 2. It can be seen that the DBNet + Res2 + AMF method can reach 84.92% and 93.94% on ICDAR2015 and F-measure on the self-built Uyghur dataset, respectively, which verifies the effectiveness of the performance of our proposed method.

In Table 2, four sets of ablation experiments are shown. Each row represents a group of experiments, and the symbols “√” and “×” in the first two columns represent the presence or absence of the module in that column, respectively. The following conclusions can be drawn from the experimental results:

1. In the first group of experiments, as shown in the second row of the table, only the Res2Net module is used for feature extraction in the text detection algorithm based on DBNet. Res2Net module improves the multi-scale representation ability at the fine-grained level, and gradually expands the receptive field in multiple convolutions to capture the details and global features on the feature map. It can be seen from Table 1, the Res2Net module significantly improves the performance of the text detection model. It improves the recall and F-measure on the ICDAR2015 dataset by 1.41% and 0.87%, respectively, and improves the precision and F-measure on the Uyghur dataset by 0.42% and 0.38%, respectively.
2. In the second group of experiments, as shown in the third row of the table, after only adding the adaptive multi-level feature fusion module, it can be seen from the experimental results that the performance gains of 0.88% and 0.26% are brought on the F values of the two data sets, respectively. Therefore, it can be seen that the AMF module can learn the method of filtering positive and negative sample conflicts in the spatial dimension during the training process, and suppress the inconsistency of multi-scale targets on the feature maps of different network levels.
3. In the third group of experiments, this paper adopts the network model of DBNet + Res2 + AMF according to the characteristics of text boxes in two datasets. Compared with using a single module, its comprehensive performance has been improved to a certain extent. In addition, compared with the baseline method, the recall and F-measure of the DBNet + Res2 + AMF model on the ICDAR2015 dataset increased by 3.24% and 1.09%. On the Uyghur dataset, its precision and F-measure are improved by 0.84% and 0.52%. It can be seen that using two improved modules at the same time is more conducive to improving the text detection performance.

### 4.4. Comparison with Classical Method

**Figure 9:**
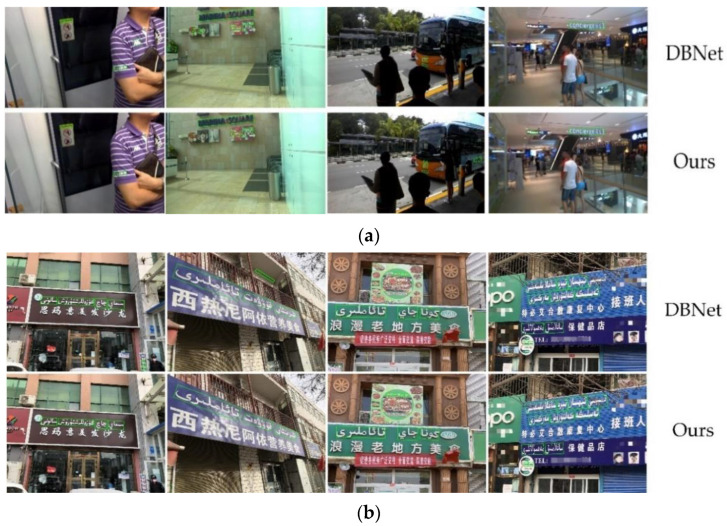
The visualization results of this method and baseline method on different datasets. (**a**) The text detection example on the public English dataset ICDAR2015; (**b**) the text detection example on self-built Uyghur dataset. (Text description in the figure (**b**) are Simayi hair salon; Xireniayi nutritious food; Romantic local food and Tebiaihesan rehabilitation center)

Based on the baseline method DBNet and the proposed improved algorithm, this paper extracts the text region on the public dataset ICDAR2015 and the self-built Uyghur dataset. The experimental visualization results are shown in Figure 9.

In columns 1 and 2 of Figure 9, the baseline method mistakenly considers the brand trademark or horizontal and vertical stripe objects similar to the font of the text as text instances. In columns 3 and columns 4, DBNet misses the detection of text instances in the text areas with small scale, fuzzy and occlusion, and the detection effect is poor. This method adopts the improved algorithm based on DBNet, and uses the 3 × 3 convolution feature group connected by multi-level hierarchical residuals to increase the receptive field, so that the feature representation has rich detail information, greatly improves the detection effect of small and medium-sized text and text area with large aspect ratio in the image, and can effectively suppress false positives. It proves that this method has better performance in text detection of Uyghur scenes.

#### 4.4.1. Text Detection on ICDAR2015 Dataset

Based on the baseline method DBNet and the proposed improved algorithm, this paper extracts the text region on the public dataset ICDAR2015, and the experimental results are shown in Table 3.

ICDAR2015 dataset is an English scene text dataset, which contains a large number of tiny and fuzzy text examples. As shown in Table 3, compared with various text detection algorithms, the F-measure of the method in this paper achieves the best performance of 84.92%, which is 1.09% higher than the baseline algorithm. The precision and recall are 88.25% and 81.84%, respectively, where the recall is only 0.16% lower than the highest PixelLink method. However, in order to distinguish text instances, the segmentation method PixelLink needs to classify the foreground and background of each pixel and predict the connection relationship between adjacent pixels, which has a high post-processing calculation cost. The method in this paper uses the differential binarization function that can be learned in training to classify the pixels directly, and its prediction of the text instance area surrounded by the text score map is more accurate. Therefore, compared with other text detection algorithms, it can simplify the post-processing process of text box inferencing and improve the detection speed. It can be seen from the table that the detection speed of the method used in this paper is generally higher than the text box regression algorithm, and 6.3 times faster than the PixelLink method.

#### 4.4.2. Text Detection on Uyghur Dataset

In this paper, EAST, DBNet and the improved algorithm proposed are used to test the self-built Uyghur scene text detection dataset. The text detection results are shown in Table 4.

Self-built Uyghur datasets mostly contain multi-scale and multi-directional perspective text. This paper uses EAST, DBNet and the improved algorithm proposed to test on the self-built Uygur scene text detection dataset. The text detection results are shown in Table 4. When DBNet + Res2 + AMF detection algorithm is used, the highest accuracy, recall, and F1 composite metrics can be achieved with 96.55%, 91.47%, and 93.94%, respectively. It can be seen that using the Res2 + AMF module improves 0.84% and 0.52% in accuracy and F-value compared to the DB + ResNet50 model, and although the DB + ResNet18 model has a greater advantage in detection speed, the DB + Res2 + AMF model has a higher performance gain of 2.17%, 2.95% and 2.58%, while the method in this paper has a greater performance gain than the single-stage regression detection algorithm EAST. In addition, because the Res2 module is an improved algorithm based on ResNet50, compared to the Resnet18 network its structure is more complex and the network depth deepens, which increases the amount of computation and also has an impact on the detection speed, but the Res2 module uses a smaller set of four-level convolution instead of the 3 × 3 convolution in the bottleneck structure, thus the algorithm in this paper is slightly lower in detection speed than the DBNet + ResNet50 algorithm 3.54 frames/second. In this paper, the convolution operation of the multi-level residual structure is introduced into the classical scene text detection algorithm. Due to the overlapping of the receptive fields, the text instance target can be captured at multiple scales, which strengthens the representation of the global semantic information by the feature map, and is effective for the detection of multi-scale Uyghur text. At the same time, the fine-grained feature capture method plays a great role in the identification of text-like objects and occluded texts.

## 5. Discussion

DBNet is a classical and commonly used text detection algorithm. In this paper, we make further optimization of the DBNet method according to the extreme scale distribution of scene text, pseudo-recall and other problems, and we adopt the fine-grained feature description algorithm Res2Net to gradually expand the perceptual field of the feature map, so that it can better adapt to the feature description of multi-scale text targets. Meanwhile, our proposed adaptive multi-level feature fusion strategy contributes to the detection accuracy of text. Due to the different resolutions of the feature maps of different scale text maps, the information conflicts in the fusion process are generated. By controlling the weights, the weights of the corresponding background levels are reset to 0, and only valid information is retained for aggregation to improve the scale invariance of features. Comparing with the classical text detection models in recent years, our proposed method effectively improves the text detection accuracy with the combination of two modules, and its comprehensive index reaches 93.94% on the Uyghur dataset and 84.92% F-value on the public dataset ICDAR2015, and still achieves 18.91 fps, faster than most algorithms, in terms of detection speed. This is a great advantage in real time detection, autonomous driving and other practical scenarios.

## 6. Conclusions

In this paper, a scene Uyghur text image dataset is constructed for the Uyghur text detection task. Secondly, the proposed text detection model consists of three parts: feature extraction, feature fusion, and text box inference. Based on the DBNet method, we improve the baseline algorithm in the two stages of feature extraction and feature fusion. To address the problems of difficult detection of multiscale adherent Uyghur text in scene images and easy false detection of objects with similar texture and text in complex backgrounds, we propose to use the Res2Net module, divide the feature description into finer modules, capture the contextual information of the target while extracting multiscale features, improve the ability of the whole model to detect and segment targets in images, and better consider the combination of different numbers of multiscale features in the residual blocks and the impact on the detection accuracy of multiscale text. Further, for the inconsistency of information in the feature fusion process of the baseline model itself, the adaptive multi-level feature fusion strategy is used to improve the scale invariance of features by adaptively learning spatial fusion weights during the training process, which can balance the information at different levels in the multi-scale feature map and filter out negative sample information. Finally, we infer the text boxes by post-processing such as differentiable binarization calculations. The method in this paper can achieve accurate localization of text boxes and has advantages in detection speed. In future work, we can embed the text detection model into the text recognition module to achieve real-time localization and recognition of scene images, which can be useful in fields such as automatic driving, paperless office, and network opinion monitoring.

## Figures and Tables

**Table 1:** The influence of residual block stratification scale.

Method	ICDAR2015				Uyghur Dataset			
	P	R	F	FPS	P	R	F	FPS
DBNet + Res2 + AMF-4s	88.25	81.84	84.92	18.91	96.55	91.47	93.94	22.81
DBNet + Res2 + AMF-6s	89.51	81.02	85.05	16.03	96.20	91.90	94.01	18.23
DBNet + Res2 + AMF-8s	90.12	82.18	85.97	12.75	96.87	92.42	94.59	12.71

**Table 2:** Ablation experiments of different modules.

Method		ICDAR2015				Uyghur Dataset			
Res2Net	AMF	P	R	F	FPS	P	R	F	FPS
×	×	89.82	78.60	83.83	22.32	95.71	91.23	93.42	26.35
√	×	89.98	80.00	84.70	20.42	96.13	91.59	93.80	24.55
×	√	89.89	80.10	84.71	21.29	96.04	91.43	93.68	24.77
√	√	88.25	81.84	84.92	18.91	96.55	91.47	93.94	22.81

**Table 3:** The results of text detection on ICDAR2015 dataset.

Method	P	R	F	FPS
EAST(VGG16 + RBOX) [15]	80.46	72.75	76.41	6.52
RRPN [12]	82.17	73.23	77.44	-
SegLink++ [13]	86.30	73.70	79.50	9.50
TextSnake [14]	84.90	80.40	82.60	1.10
PixelLink [22]	85.50	82.00	83.87	3.00
TextField [24]	84.30	83.90	84.10	1.80
FCENet [18]	84.20	85.10	84.60	-
DBNet + ResNet18	88.03	78.01	82.72	38.93
DBNet + ResNet50	89.82	78.59	83.83	22.32
DBNet + Res2	89.98	80.00	84.70	20.42
DBNet + Res2 + AMF	88.25	81.84	84.92	18.91

**Table 4:** The results of text detection on Uyghur dataset.

Method	P	R	F	FPS
EAST(VGG16 + RBOX)	82.68	88.44	85.46	1.16
DBNet + ResNet18	94.38	88.52	91.36	45.16
DBNet + ResNet50	95.71	91.23	93.42	26.35
DBNet + Res2	96.13	91.59	93.80	25.36
DBNet + Res2 + AMF	96.55	91.47	93.94	22.81

## Data Availability

The ICDAR2015 public dataset used in this article is available at http://rrc.cvc.uab.es/?ch=4. (accessed on 1 May 2022), The self-built scene Uyghur text image dataset used in this paper has not been made public and it belongs to the Academy and will be released soon.
